# Effectiveness of a Telephonic Aging Brain Care Model for Medicaid Home and Community Services for Dementia Patients and Their Caregivers

**DOI:** 10.1111/jgs.70146

**Published:** 2025-10-04

**Authors:** Malaz A. Boustani, Steven R. Counsell, Anthony Perkins, Abdelfattah Alhader, Kathryn I. Frank, Diana P. Summanwar, Karen L. Fortuna

**Affiliations:** ^1^ Department of Medicine Indiana University School of Medicine Indianapolis Indiana USA; ^2^ Center for Health Innovation and Implementation Science Indiana University School of Medicine Indianapolis Indiana USA; ^3^ Department of Biostatistics and Health Data Science Indiana University School of Medicine Indianapolis Indiana USA; ^4^ Department of Physiology and Biochemistry Faculty of Medicine, Jordan University of Science and Technology Irbid Jordan; ^5^ Department of Family Medicine Indiana University School of Medicine Indianapolis Indiana USA; ^6^ Department of Community and Family Medicine, Geisel School of Medicine at Dartmouth Lebanon New Hampshire USA

**Keywords:** caregiver stress, caregiver support, dementia

## Abstract

**Objectives:**

The primary purpose of the present study was the implementation and evaluation of the ABC Community program, a community‐based and telephonically administered version of the Aging Brain Care model delivered by Area Agencies on Aging (AAAs) staff.

**Design:**

This study employed a prospective pre‐post implementation design with pre‐specified effectiveness and fidelity goals, with the main outcome measure being the total score of the Health Aging Brain Care (HABC) Monitor at 3‐ and 6‐month follow‐up. The HABC Monitor has demonstrated excellent reliability and validity in monitoring and measuring the burden of dementia symptoms and the quality of life and stress of the informal caregivers.

**Results:**

The program served 422 Medicaid Home and Community‐Based Services participants living with dementia and their caregivers. Participants' mean age was 78 years, with 67% identifying as female and 30% as belonging to minority groups, including 6% Hispanic or Latino and 28% Black or African American. In comparison to baseline, the total score of the HABC Monitor decreased from 24.6 to 15.4 at 6 months, representing a 37% reduction with an effect size of 0.64 standard deviation (*p* value < 0.001). Approximately 46% of informal caregivers who had at least mild burden at baseline had no such burden at 6 months, and 92% of those who had no stress at baseline remained burden‐free at 6 months.

**Conclusion:**

The ABC community program might be a scalable collaborative dementia care model targeting socially vulnerable people living with dementia.

## Graphical abstract

The conceptual Model for ABC Community program starts with measuring the competncies of the caregiver in coping with dementia related disabilities followed by developing a peornsalized coaching plan and monitoring the effectivenss of such a plan.

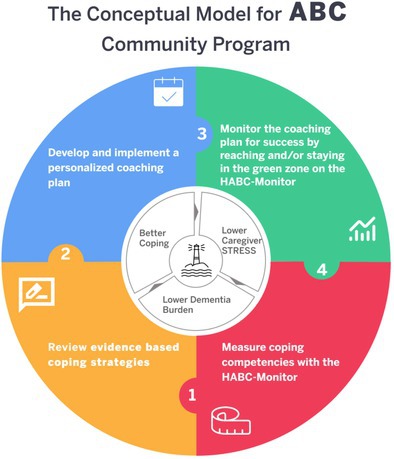

**Summary**

- Key points
  ○ The ABC Community program, a community‐based and telephonically administered version of the Aging Brain Care model, was effective in reducing informal caregivers' stress and improving their overall well‐being.
  ○ Utilizing Area Agencies on Aging to deliver the ABC Community program made the program scalable.
  ○ The ABC Community program has a sustainable impact.
- Why does this paper matter?
  ○ The ABC Community program is a potentially scalable model to reduce the burden of informal caregivers of people living with dementia.

## 1 Introduction

Over 11 million family members and other informal caregivers are dedicating approximately 18 billion hours to caring for people living with dementia (PLWD) in the United States of America [1, 2]. These informal caregivers are experiencing chronic physical and psychological strain [3, 4] with little or no coaching in dementia care [5]. Over the past two decades, several collaborative dementia care models have been developed to meet the complex biopsychosocial needs of PLWD and their informal caregivers [6, 7, 8, 9, 10, 11, 12]. These models deliver coordinated and personalized dementia care that includes coaching the informal caregivers on how to manage the cognitive, physical, behavioral, and psychological disabilities of their loved ones and their own stress.

One of the collaborative care models is the Aging Brain Care (ABC) model [6, 13]. The ABC model uses an interdisciplinary clinical team based in a healthcare delivery system to develop, deliver, and monitor the effectiveness of a personalized dementia care plan. A randomized controlled trial and several implementation studies found that the ABC model is effective in reducing the behavioral and psychological symptoms related to dementia and reducing informal caregiver stress [6, 8, 13]. Indiana University (IU) was awarded funding through a 3 years (August 2020—July 2023) cooperative agreement under the Administration for Community Living (ACL) Alzheimer's Disease Programs Initiative (ADPI) intended to support and promote the development and expansion of dementia‐capable home‐ and community‐based service (HCBS) systems in states and communities. IU partnered with five Indiana Area Agencies on Aging (AAA) and the University of Indianapolis to design, implement, and evaluate a revised version of the original ABC model called the ABC Community program, where the dementia care plan is designed, delivered, and monitored by community‐based dementia care coaches who are hired by the AAAs and trained by IU dementia care experts. Other organizational and agency partners included the Indiana Association of Area Agencies on Aging, the Alzheimer's Association of Greater Indiana Chapter, and the Indiana Family and Social Services Administration's Division of Aging. This paper evaluates the effectiveness of the ABC Community version of the ABC model delivered telephonically by AAA staff.

## 2 Methods

### 2.1 Study Design

We employed a prospective pre‐post implementation design aimed at evaluating the effectiveness of a community‐based version of the ABC model delivered telephonically by AAA staff with pre‐specified fidelity and effectiveness goals. The main outcome measure was the total score of the Healthy Aging Brain Care (HABC) Monitor at 3‐ and 6‐month follow‐up. The HABC Monitor (see Supporting Information S1) has demonstrated excellent reliability and validity in monitoring and measuring the severity of dementia symptoms and the competency of the informal caregiver's ability to cope with the cognitive, functional, behavioral, and psychological disability related to dementia [14]. The HABC‐Monitor includes 31 items. Each item has the same response options (0, 1, 2, or 3) corresponding to the frequency of a target symptom or problem within the past 2 weeks according to the informal caregiver [14]. The IU—Human Subjects Office determined that this project was being conducted as a service and quality improvement project rather than research and thus did not require Institutional Review Board review.

### 2.2 Participants Enrollment

Eligible participants for the ABC Community program included PLWD aged 60 or older enrolled in Indiana's Medicaid HCBS Aged and Disabled Waiver (ADW) program. ADW eligibility criteria included being aged, blind, or otherwise disabled; having an income at or below 300% of the Supplemental Security Income amount; and meeting Indiana Medicaid nursing facility level of care as indicated by dependence in multiple activities of daily living. ADW participants, although qualified for long‐term nursing home residence, chose instead to reside in community settings with support from waiver services and supports. Participants and their informal caregivers were enrolled in this study of ABC Community through referrals from waiver service coordinators at each of the AAAs, often by warm hand‐off to the care coach within the same AAA. Participants and/or their caregivers could have declined the program when offered by the waiver service coordinator or any time thereafter.

### 2.3 Program Care Coach Recruitment and Training

The ABC Community version of the ABC model was delivered by 5 full‐time ABC Community Care Coaches. The Care Coaches were employed by the five AAAs (Planning and Service Areas 2, 3, 6, 8, and 11), representing 34 of 92 Indiana counties. Care Coaches were required to have at least a high school diploma, an associate degree, 2 years of related experience, and/or Community Health Worker Certification. AAAs were provided the needed support in recruiting ABC Community Care Coaches, including the provision of a job description template. Each potential candidate completed a survey about attitudes toward older adults during the interview process and responded to case discussions during a virtual interview. Each Care Coach was supported by a Project Site Coordinator designated by the respective AAA and was fully supported by grant and cost sharing/match funds throughout the project period.

The 5 ABC Community Care Coaches and their corresponding coordinators completed an initial intensive 10 half‐days virtual ADRD care training (40 h total), followed by 4 half‐day virtual booster trainings (8–12 h total) every 6 months. Trainings were provided by IU dementia care experts and based on the training program of the ABC model [15]. ABC Community Care Coaches were also provided with ongoing guidance and support through virtual dementia care case conferences held weekly for the first 6 months, then twice monthly. Dementia care case conference guidelines and a presentation template were developed to help ensure optimal shared learning and support of the ABC Community Care Coaches.

### 2.4 The Services of the ABC Community Program

Delivering the Caregiver Stress Prevention Bundle is the main component of the ABC Community program. The four key components of the bundle are (1) caregiver counseling, education, and referral—including communication skills, behavioral symptom management training, coping skills, home safety, driving, and financial and legal planning; (2) development of crisis plans—including an “if‐then” plan for home safety concerns and future hospital admissions or emergency department visits; (3) weekly respite care—scheduled 8 h per week time off caregiving responsibilities; and (4) monthly support group participation—with concurrent activities for the person with ADRD at the same location. The efficacy and effectiveness of the bundle were demonstrated by previous studies [6, 8, 13]. Working closely with corresponding AAA waiver service coordinators, ABC Community Care Coaches enrolled participant and informal caregiver dyads into the program. The Care Coaches subsequently made telephone contacts (home visits were precluded due to the COVID pandemic) as determined by dementia symptoms and informal caregiver burden levels. Each participant received a minimum of monthly contacts during the first 3 months. Subsequent contacts were adjusted to quarterly intervals unless higher needs were identified. ABC Community Care Coaches assessed dementia symptoms and informal caregiver burden at each contact using the HABC Monitor [14]. If the HABC monitor score was over a certain cut point (representing high caregiver burden, see Supporting Information S1), more intensive counseling and support were provided by the Care Coach through weekly contacts for 8 weeks, after which the participant and informal caregiver dyad were reassessed. Handouts from the *Caregiver Resource Handbook* were mailed to informal caregivers to augment counseling and education [13]. Finally, ABC Community Care Coaches presented challenging cases at the Dementia Care Case Conferences, during which they received input from dementia care experts and exchanged effective intervention strategies to help address common issues.

### 2.5 Program Evaluation

Process and outcome measures were collected by the ABC Community Care Coaches at one, two, three, and 6 months using an online REDCap survey tool. Process or fidelity measures were determined as the percentage of participants receiving each of the four key components of the caregiver stress prevention bundle. Fidelity refers to the degree to which an intervention is delivered as intended and is particularly important in this study to ensure consistent implementation of the bundle across a large, community‐based setting.

Outcome measures included a reduction in dementia symptoms related burden and informal caregiver stress, as captured by the HABC Monitor [14]. The HABC Monitor exhibits adequate item and scale score variability, low item missing rates, and no problematic floor or ceiling effects. The internal consistency of the HABC‐M scales is high, ranging from 0.73 to 0.92, and the Monitor demonstrated acceptable construct validity with respect to the caregiver‐reported Neuropsychiatric Inventory (NPI) assessment of patient symptoms; the caregiver distress of the NPI, and the clinician‐based staging of dementia [14]. The HABC Monitor is also sensitive to change, as indicated by significant differences between NPI ‘reliable change’ groups on the mean change scores of the HABC‐M scale [14].

**FIGURE 1:**
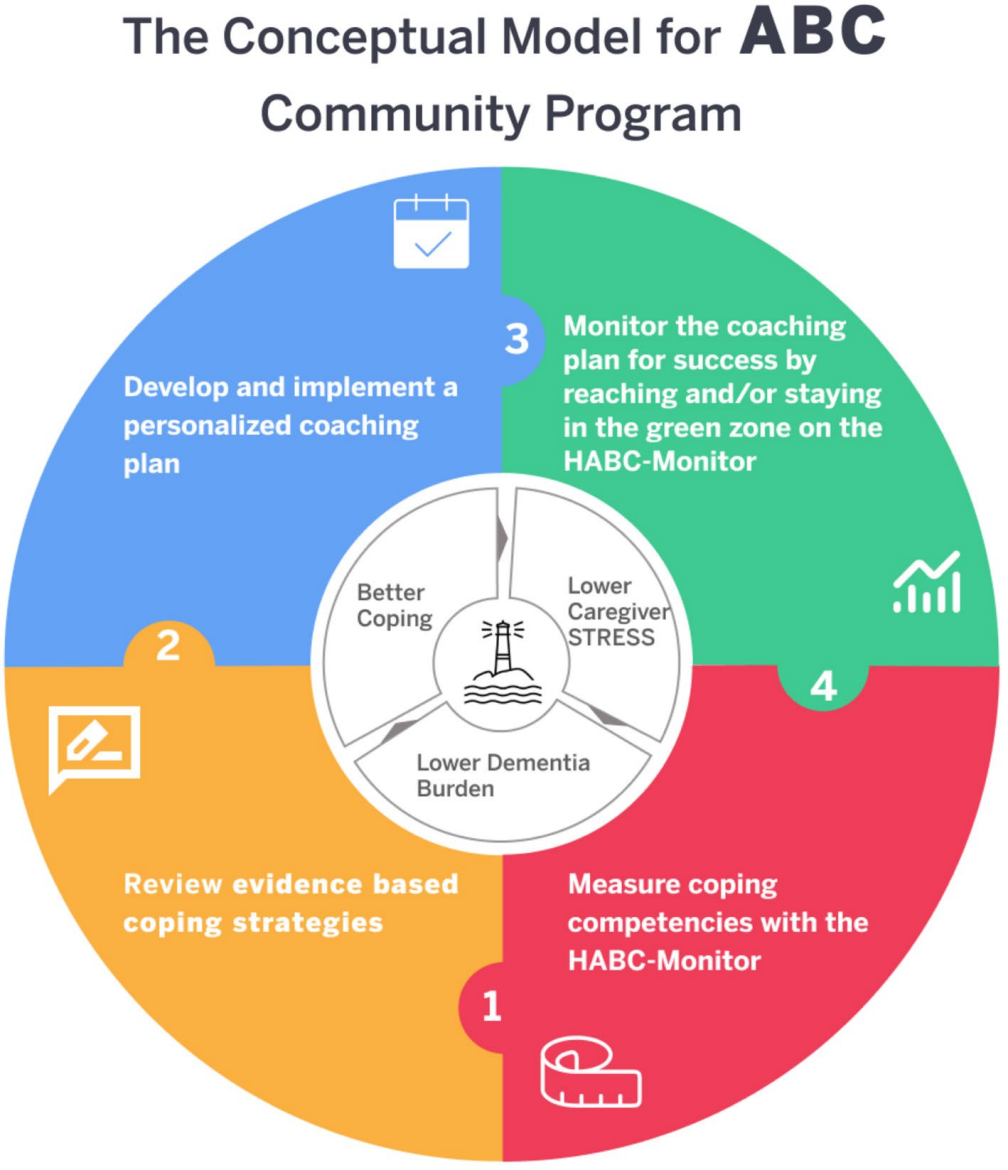
The conceptual model for ABC Community program. Abbreviations: ABC, Aging Brain Care; HABC, Healthy Aging Brain Care.

Baseline data were gathered from all participants prior to the initiation of the program using the HABC Monitor. Follow‐up assessments using the HABC Monitor were conducted at 1, 2, 3 months, and at 6 months post‐implementation. Descriptive statistics were employed to summarize demographic characteristics of the participants and baseline measurements. We used a repeated measures model to analyze the trajectory of the HABC Monitor over time with a fixed effect for time. An unstructured variance–covariance matrix was used in the models to account for correlated measurements within patients. We also used a similar model that included fixed effects for time, attrition at 6 months, and the interaction between time and 6‐month attrition to determine the effect of attrition on the HABC Monitor over time. The total score of the HABC Monitor ranges from 0 to 93 points, with a score below 15 indicating the absence of caregiver stress. A change of at least five points in the total HABC Monitor score is considered a clinically meaningful improvement. We employed the McNemar chi‐squared test to analyze paired, dichotomous data on both the reduction and prevention of informal caregiver stress. All analyses were performed using SAS v9.4. An alpha level of 0.05 was set for determining statistical significance (Figure 1).

## 3 Results

### 3.1 Population Characteristics

Table 1 shows the overall population characteristics of the ABC Community program. Among the 422 participants, the mean age was 78 years, with 67% identified as female and 30% as minority groups, including 6% Hispanic or Latino and 28% Black or African American. Among the 422 informal caregivers included, 80% were female and 28% minorities, with 4% Hispanic or Latino and 28% Black or African American. Additionally, among program participants, 51% were parents of their informal caregivers, 33% were spouses, and 16% were classified as other types of informal caregivers. A significant majority, 82%, lived with the informal caregiver, whereas 17% lived alone, and only 1% resided with a non‐caregiver.

**TABLE 1:** Demographic characteristics and dementia symptoms related burden of people living with dementia and their informal caregivers at the time of their initial enrollment in the ABC Community program.

	Patient (*n* = 422)	Caregiver (*n* = 422)
Mean age (SD)	78.4 (9.5)	58.1 (14.0)
Female, *n* (%)	282 (66.8)	339 (80.3)
Race, *n* (%)		
American Indian or Alaskan Native	1 (0.2)	0 (0.0)
Asian or Asian American	1 (0.2)	1 (0.2)
Black	118 (28.0)	117 (27.7)
White	295 (69.9)	297 (70.4)
Unknown/not reported	7 (1.7)	7 (1.7)
Ethnicity, *n* (%)		
Hispanic or Latino	24 (5.7)	16 (3.8)
Not Hispanic or Latino	395 (93.6)	404 (95.7)
Unknown/not reported	3 (0.7)	2 (0.5)
Living arrangement at enrollment, *n* (%)		
Lives alone, has identified caregiver	71 (16.8)	
Lives with a caregiver	345 (81.8)	
Lives with someone who is not a caregiver	6 (1.4)	
Relationship to caregiver, *n* (%)		
Spouse or partner	140 (33.2)	
Parent	216 (51.2)	
Other caregiver	66 (15.6)	
HABC Monitor (mean, SD or *N*, and %)		
Mean total score and SD	24.6 (14.4)	
No burden (HABC score < 15)	118 (28%)	
Mild burden (HABC score 15–23)	98 (23%)	
Moderate burden (HABC score 24–33)	99 (24%)	
Severe burden (HABC score > 33)	107 (25%)	

Abbreviations: HABC, healthy aging brain care; SD: standard deviation.

Table 1 also reveals the baseline HABC scores, with means presented alongside standard deviations (SD). The overall mean total score was 24.6 (SD = 14.4). The distribution of severity of dementia‐related burden showed 118 individuals (28%) were in the no burden range (< 15), 98 (23%) had mild burden (15–23), 99 (24%) presented with moderate burden (24–33), and 107 (25%) were categorized as having severe dementia symptoms‐related burden (> 33).

### 3.2 Fidelity and Process Measures

Table 2 presents the percentage of participant/caregiver dyads who received each of the four key components of the caregiver stress prevention bundle: (a) 93% at 1 month to 84% at 6 months received counseling and education; (b) 95% at 1 month to 99% at 6 months received a crisis plan; (c) 69% at 1 month to 74% at 6 months received regularly scheduled time‐off caregiving tasks; and (d) 19% at 1 month to 21% at 6 months attended a support group. The results demonstrate the implementation exceeded the anticipated fidelity targets (25%, 50%, 10%, and 25%, respectively), except for attending a support group. Over 6 months (see Table S1), the care coaches conducted an average of 8.3 visits per participant with a SD of 5.3 visits (Median of 6.5 visits with 25% lower quartile of 2 visits and 75% high quartile of 26 visits). In Table S2, we report the percentage of participants who their informal caregiver received coaching for each sub item of the Caregiver Stress Prevention Bundle with 52% of them receiving coping coaching for repetitive behaviors, 48% for depression and anxiety, 47% for stress, 40% for agitation or aggression, and 39% for sleep disturbances.

**TABLE 2:** Participant–caregiver dyads who received key components of the caregiver stress prevention bundle over time.

	Targets^a^	1 month (*n* = 301)	2 months (*n* = 257)	3 months (*n* = 261)	6 months (*n* = 222)
Counseling, *n* (%)	25%	280 (93.05)	229 (89.1)	244 (93.5)	186 (83.8)
Crisis plan, *n* (%)	50%	285 (94.7)	249 (96.9)	257 (98.5)	220 (99.1)
Time off, *n* (%)	10%	209 (69.4)	195 (75.9)	189 (72.5)	165 (74.3)
Support group *n* (%)	25%	56 (18.6)	51 (19.8)	54 (20.7)	47 (21.2)

*Note:* Color code: Green color indicates that the anticipated target was met at 6 months and red color indicates that the anticipated target was not met at 6 months.

^a^ Target was defined as the minimum fidelity goals for the percentage of enrollees who would receive each component of the caregiver stress prevention bundle at 6 months.

### 3.3 Outcome Measures

At the 6‐month follow‐up, HABC Monitor data were missing for 204 out of 422 participants due to the following reasons: 58 missed the visit, 8 changed informal caregivers, 1 due to participant request, 30 due to informal caregiver requests, 17 cases of non‐adherence by informal caregivers and/or participants, 1 lost to follow‐up, 34 deaths, 11 assisted living placements, 29 nursing home placements, and 15 for other reasons.

Table 3 illustrates the long‐term impact of the ABC Community program. The mean total HABC Monitor score among the group who completed a 6‐month assessment (*n* = 218) dropped from 24.6 at baseline to 19.1 at 1 month, 16.3 at both 2 and 3 months, and 15.4 at 6 months. The percentage decrease from baseline was 22% at 1 month, 34% at 2 and 3 months, and 37% at 6 months. The change over time was similar for the group who completed the 6‐month assessment and those with attrition at 6 months. Those who left the program prior to 6 months had significantly higher scores at baseline than those who completed the 6‐month assessment. These findings indicate a reduction in HABC Monitor over the first 2 months that was sustained through 6 months.

**TABLE 3:** Longitudinal changes in HABC Monitor total scores and percent change from baseline at 1, 2, 3, and 6 months^a^.

	Group with no 6 month visit		Group with 6 month visit		Overall	
	*N*	Mean (SD)	*N*	Mean (SD)	*N*	Mean (SD)
Baseline	204	26.9 (15.5)	218	22.5 (13.0)	422	24.6 (14.4)
1 Month	110	21.6 (13.3)	187	17.5 (12.0)	297	19.1 (12.6)
2 Month	74	20.5 (11.8)	182	14.5 (9.8)	256	16.3 (10.8)
3 Month	59	19.9 (13.4)	200	15.3 (11.5)	259	16.3 (12.1)
6 Month	0		218	15.4 (13.4)	218	15.4 (13.4)

*Note:* A model with time, attrition at 6 months, and the interaction of time and attrition revealed that there was no significant interaction (*p* = 0.854) between time and attrition signifying the change over time was similar by attrition status. A separate model with just fixed effects for attrition and time (no interaction) showed those without a 6‐month visit had significantly higher scores (*p* = 0.031) and similar time effects in the model without attrition status. Stratifying the analysis by those who dropped out vs. those that stayed in the study, show similar results.

Abbreviations: HABC, Healthy Aging Brain Care; SD, standard deviation.

^a^ Statistical comparison of log‐transformed scores over time (log [score + 1]) revealed that scores at 6 months were significantly lower than those at 1 month (*p* < 0.001) and baseline (*p* < 0.001). Scores at 3 months were significantly lower than those at 1 month (*p* < 0.001). Additionally, scores at 2 months were significantly lower than those at 1 month and baseline (*p* < 0.001).

Furthermore, the effectiveness of the ABC Community program was evaluated at the individual level. Of those informal caregivers who had at least mild dementia symptoms related burden at baseline (*n* = 145), 67 (46%) showed significant improvement and moved to the “Green Zone” (defined as HABC Monitor score < 15), whereas 78 (54%) remained in the “Red Zone” (*p* < 0.001). Additionally, the effectiveness of ABC Community in preventing dementia symptoms related burden was assessed among informal caregivers who were initially in the “Green Zone,” that is, with a HABC Monitor score < 15 at baseline (*n* = 73). Of these, 67 (92%) remained in the “Green Zone” at 6 months, which was significantly (*p* < 0.001) greater than those who started in the “Red Zone”, whereas 6 (8%) moved above the threshold to the “Red Zone.”

## 4 Discussion

Our implementation study showed that the ABC Community program was effective in reducing the burden of cognitive, physical, behavioral, and psychological symptoms related to dementia and informal caregiver stress with moderate effect sizes at 3 months (0.58 SD of the total score of the HABC Monitor) and 6 months (0.64 SD of the total score of the HABC Monitor). Approximately 46% of informal caregivers who had at least a mild burden at baseline had no such burden at 6 months, and 92% of those who had no burden at baseline remained burden‐free at 6 months. Such a positive effect was mediated by a high fidelity of delivering the Caregiver Stress Prevention Bundle, with 84% receiving counseling and education, 99% completing one or more crisis plans, 74% having regular informal caregiver time off, and 21% of caregivers attending a support group at 6 months.

The effectiveness of the ABC Community program in reducing dementia symptoms‐related burden aligns with previous studies highlighting the positive impact of collaborative dementia care models. For example, the effect of the ABC Community program was similar to the effect of the original ABC model that was delivered by an interdisciplinary team of clinicians (a community health worker, a social worker, a nurse, a geriatrician, and an administrator) employed by an integrated health care delivery system [13]. In 2015, we evaluated the implementation of the ABC Model among 378 PLWD in Central Indiana with a mean age of 79.8 years (a similar age to the current ABC Community study) and a SD of 8.2 years. At baseline, the mean HABC Monitor score among the participants was 23.7 points with a SD of 18.1 in comparison to a mean HABC Monitor score of 24.6 with a SD of 14.4 among the participants in the current ABC Community program. In comparison to the 9.2 points reduction in HABC Monitor score at 6 months observed in the ABC Community program, there was an average of 5.8 points reduction at 12 months in the ABC Model [13]. Furthermore, the effectiveness of the ABC Community program was similar to other collaborative dementia care models delivered by healthcare systems such as the Care Ecosystem at the University of California in San Francisco, the Dementia Care program at the University of California in Los Angeles, the Integrated Memory Care Clinic at Emory University, and the MIND at Home program at Johns Hopkins [16].

In 2024, the Center for Medicare and Medicaid Services launched the GUIDE program as an alternative payment model to deliver the collaborative dementia care model across 400 organizations in the United States [17]. Although the evaluation of the ABC Community program was completed prior to July 2024, when the GUIDE program started in Indiana, the PLWD who were enrolled in the ABC Community program, unless a member of a Medicare Advantage Plan, would likely have met eligibility criteria for GUIDE.

Moreover, the importance of culturally competent care models is highlighted by the representation of minority groups in our study, which showed 28% of participants identifying as Black or African American. Research shows that while ethnic and minority populations generally have higher rates of dementia, they nevertheless have lower access to caregiving services and information [18, 19]. In particular, individuals from minority backgrounds may encounter cultural and linguistic barriers when communicating with healthcare professionals, which can lead to compounded caregiver burdens [19]. These barriers often make it harder for informal caregivers who may feel disconnected from available support systems. Non‐healthcare professionals employed by community‐based organizations such as in the ABC Community program may be better able to connect and build trusting relationships by which to support minority elders and their caregivers.

Our evaluation of the ABC Community program had several limitations. First, the participants were enrolled in Indiana's Medicaid ADW program and, as such, were low‐income and met the nursing facility level of care. This high‐need population may be especially helped by added resources and supports aimed at relieving informal caregiver stress. Results of this study may not be applicable to other less vulnerable populations. Second, the COVID‐19 pandemic and associated public health mitigation efforts necessitated changing in‐person trainings to virtual and home visits to telephone contacts; and participation in support groups was less than target. Reductions in dementia symptoms burden by ABC Community program, however, were comparable to those observed with the original ABC model, which was based on in‐person training and home visits. Furthermore, telephone‐based delivery of the program has continued post‐pandemic with similar acceptance and engagement levels. Third, the ABC Community program was funded by the ADPI of the ACL and Department of Health and Human Services. This ADPI is not a research program. It is a service program supported by a grant from ACL and in partnership with the grantee providing cost share/match (in this case the 5 AAAs provided required matching funds). As such, we did not track “recruitment rate” or how many eligible PLWD and/or caregivers were offered the program. Lastly, we have HABC Monitor data at 6 months for only 218 dyads of the 422 initially enrolled in the program. Such an attrition may have induced bias. However, we have conducted a sensitivity analysis to address the attrition bias by examining the interaction between time and attrition. This interaction was not significant, indicating that change over time of total HABC Monitor scores was similar between those who completed the 6‐month assessment and those who withdrew or otherwise were not assessed at 6 months.

In conclusion, we successfully implemented a collaborative dementia care program delivered telephonically by AAAs targeting socially vulnerable and diverse PLWD and their informal caregivers. Following the successful completion of this project, a service grant from the Indiana Family and Social Services Administration (FSSA) allowed ABC Community to continue with the original 5 AAAs and expand to involve 5 additional AAAs. Upon conclusion of grant funding and beginning in January 2025, the ABC Community program was sustained through a partnership of 8 AAAs with IU and two funding sources: (a) in the service category of “caregiver training” under the National Family Caregiver Support Program (Older Americans Act Title III‐E), and (b) as optional caregiver training and support for informal caregivers of PLWD enrolled in the state‐funded Community and Home Options to Institutional Care for the Elderly and Disabled (CHOICE) program.

## Author Contributions

Concept and design: Malaz A. Boustani, Steven R. Counsell, and Kathryn I. Frank. Acquisition, analysis, or interpretation of data: Malaz A. Boustani, Steven R. Counsell, and Kathryn I. Frank, Anthony Perkins, and Abdelfattah Alhader. Drafting of the manuscript: Malaz A. Boustani, Steven R. Counsell, Abdelfattah Alhader, Diana P. Summanwar, and Karen L. Fortuna. Critical review of the manuscript for important intellectual content: All authors. Statistical analysis: Anthony Perkins and Malaz A. Boustani. Obtained funding: Steven R. Counsell and Malaz A. Boustani. Administrative, technical, or material support: Malaz A. Boustani, Steven R. Counsell, and Kathryn I. Frank. Supervision: Malaz A. Boustani and Steven R. Counsell.

## Disclosure

Sponsor's role: The project was conducted independently of its sponsors. There was no sponsor involvement in the design, collection, analysis, and interpretation of the data, in the writing of the report, or in the decision to submit for publication.

## Conflicts of Interest

Dr. Boustani serves as a chief Scientific Officer and co‐Founder of BlueAgilis and the Chief Health Officer of Mozyne Health Inc. He has equity interest in Blue Agilis Inc. and Mozyne Health Inc. He gave up his shares with DigiCare Realized (the company was folded early in 2025). He sold his equity in Preferred Population Health Management LLC and My Shift Inc. (previously known as Rest Up LLC). He serves as an advisory board member or consultant for NeuroX Inc., Eli Lilly and Co., Eisai Inc., Merck & Co. Inc., Biogen Inc., and Genentech Inc. These conflicts have been reviewed by Indiana University and have been appropriately managed to maintain objectivity. The other authors declare no conflicts of interest.

## Linked Article

This publication is linked to a related Editor’s note by Michael L. Malone. To view this article, visit https://doi.org/10.1111/jgs.70145.

## Supporting information


**Table S1:** This table presents data on the frequency and distribution of visits made by community care coaches during the study period.
**Table S2:** This table details the various components of the caregiver stress prevention bundle, including strategies employed to support caregivers.
**Supporting Information S1:** Healthy Aging Brain Care (HABC) Monitor—caregiver version: This comprehensive tool is designed for caregivers to assess and document changes in the cognitive, functional, and emotional well‐being of their loved ones. It also includes items for monitoring the caregivers' own health and stress levels.
