# Supplementary material for: Effectiveness of a Telephonic Aging Brain Care Model for Medicaid Home and Community Services for Dementia Patients and Their Caregivers
Source: J Am Geriatr Soc. 2025 Oct 4;74(2):547–54. doi: 10.1111/jgs.70146 (PMC12911546; doi:10.1111/jgs.70146)
Supplement: Supplementary file 1 — Table S1: This table presents data on the frequency and distribution of visits made by community care coaches during the study period. Table S2: This table details the various components of the caregiver stress prevention bundle, including strategies employed to support caregivers. Supporting Information S1: Healthy Aging Brain Care (HABC) Monitor—caregiver version: This comprehensive tool is designed for caregivers to assess and document changes in the cognitive, functional, and emotional well‐being of their loved ones. It also includes items for monitoring the caregivers' own health and stress levels. [file JGS-74-547-s001.pdf]

Supplemental Table 1. Number of Visits by ABC Community Care Coaches

	Overall (n=422)		Red Zone (n=304)		Green Zone (n=118)		P-value
	Mean (SD)	Median (Range)	Mean (SD)	Median (Range)	Mean (SD)	Median (Range)	
**3 Months**							
# Required Visits	3.0 (1.2)	3 (1, 4)	2.9 (1.2)	3 (1, 4)	3.1 (1.1)	4 (1, 4)	0.080
# Extra Visits	3.1 (2.7)	2 (0, 11)	3.8 (2.7)	3 (0, 11)	1.2 (1.7)	1 (0,8)	<0.001
# Total Visits	6.0 (3.1)	5 (2, 15)	6.7 (3.2)	6 (2, 15)	4.3 (1.9)	4 (2, 12)	<0.001
**6 Months**							
# Required Visits	3.5 (1.5)	4 (1, 5)	3.4 (1.5)	4 (1, 5)	3.7 (1.5)	5 (1, 5)	0.026
# Extra Visits	3.8 (4.7)	3 (0, 21)	5.9 (4.8)	5 (0, 21)	2.0 (3.2)	1 (0, 19)	<0.001
# Total Visits	8.3 (5.3)	6.5 (2, 26)	9.3 (5.5)	8 (2, 26)	5.8 (3.5)	5 (2, 23)	<0.001

Supplemental Table 2. Caregiver Stress Prevention Bundle: Protocols, Crisis Plans, Caregiver Respite, and Caregiver Support Groups within the first 6 Months for Everybody

	Overall (n=422)	Red Zone (n=304)	Green Zone (n=118)
**Protocols n (%)**			
Stress	197 (46.7)	151 (49.7)	46 (39.0)
Exercise	50 (11.8)	28 (9.2)	22 (18.6)
Communication	118 (28.0)	90 (29.6)	28 (23.7)
Legal and Financial	44 (10.4)	35 (11.5)	9 (7.6)
Physical Health	94 (22.3)	66 (21.7)	28 (23.7)
Depression/Anxiety	204 (48.3)	151 (49.7)	53 (44.9)
Repetitive Behavior	220 (52.1)	171 (56.2)	49 (41.5)
Aggression/Agitation	168 (39.8)	131 (43.1)	37 (31.4)
Mobility: Wandering and Shadowing	86 (20.4)	70 (23.0)	16 (13.6)
Mobility: Balance/Falls	147 (34.8)	118 (38.8)	29 (24.6)
Bathing	142 (33.6)	118 (38.8)	24 (20.3)
Dressing	24 (5.7)	17 (5.6)	7 (5.9)
Personal Care: Meals/Inappropriate Eating Behavior & Dental Care	118 (28.0)	91 (29.9)	27 (22.9)
Toileting/Incontinence	38 (9.0)	27 (8.9)	11 (9.3)
Sleep Disturbance	165 (39.1)	121 (39.8)	44 (37.3)
Delusions/Hallucinations/Paranoia	103 (24.4)	80 (26.3)	23 (19.5)
Delirium: Prevent Delirium in the Hospital	3 (0.7)	2 (0.7)	1 (0.8)
Delirium: Post Hospitalization Medication Reconciliation	1 (0.2)	1 (0.3)	0 (0.0)
# Protocols, Median (Range)	5 (0. 9)	5 (0, 9)	4 (0, 8)
**Crisis Plans n (%)**			
Hospital Visits	325 (77.0)	241 (79.3)	84 (71.2)
ED Visits	295 (69.9)	217 (71.4)	78 (66.1)
Home Safety	305 (72.3)	224 (73.7)	81 (68.6)
Driving	236 (55.9)	180 (59.2)	56 (47.5)
Medication Adherence	272 (64.4)	201 (66.1)	71 (60.2)
Financial Planning	191 (45.3)	133 (43.8)	58 (49.2)
Legal Planning	148 (35.1)	101 (33.2)	47 (39.8)
Guardianship	23 (5.4)	19 (6.2)	4 (3.4)
POST	53 (12.6)	32 (10.5)	21 (17.8)
Cancer Screening	13 (3.1)	10 (3.3)	3 (2.5)
Elder Abuse	4 (1.0)	1 (0.3)	3 (2.5)
**Caregiver Respite/Scheduled Time Off n (%)**			
Yes	330 (78.2)	231 (76.0)	99 (83.9)
**Quantity of Caregiver Respite/Scheduled Time Off n (%)**			
8 or more consecutive hours per week	144 (43.6)	93 (40.3)	51 (51.5)
8 or mor non-consecutive hours per week	127 (38.5)	92 (39.8)	35 (35.4)
Under 8 hours per week	59 (17.9)	46 (19.9)	13 (13.1)
**Caregiver Support Group n (%)**			
Yes	100 (23.7)	71 (23.4)	29 (24.6)
**Support Group Type n (%)**			

Monthly caregiver/participant support group	14 (14.0)	9 (12.7)	5 (17.2)
Less than monthly caregiver/participant support group	7 (7.0)	4 (5.6)	3 (10.3)
Monthly caregiver only support group	46 (46.0)	35 (49.3)	11 (37.9)
Less than monthly caregiver only support group	33 (33.0)	23 (32.4)	10 (34.5)

Healthy Aging Brain Care (HABC) Monitor – Caregiver Version

Dear [Caregiver]: ____

Your _____________________ is a patient of the ______________________. We would appreciate it if you would complete the attached HABC Monitor form. Your answers are important in helping us with the ongoing evaluation and treatment of ______________________. In particular, we appreciate your help in monitoring any changes in your ________________________'s memory, mood, behaviors, and day-to-day activity. We are also concerned about your overall health.

When you are completing this form, please keep in mind the following:

1. Please mark each item based on your first reaction – evidence of actual change is not as important as your gut instinct.

2. When determining whether your loved one has a problem with an activity, answer affirmatively only if:

   - Your loved one has difficulty with the activity AND he/she is distressed by that; OR
   - Your loved one has difficulty with the activity AND you are distressed by that.

   For example, if your loved one is unable to do household chores, but you have hired someone to do the housework AND both you and your loved one are comfortable with that arrangement, then it's not a problem.

3. Rate the frequency of the symptoms over the past two weeks using a scale of:

   - Not at all (0-1 day)
   - Several days (2-6 days)
   - More than half the days (7-11 days)
   - Nearly every day (12 -14 days)

4. What is your date of birth? ______________

5. What is your race? White Black Asian Hispanic Other _____________

6. How many years of education did you complete? ________________

7. How well do you know the patient? ☐ Not at all ☐ Somewhat well ☐ Well ☐ Very well

Over the past **two weeks**, how often did **your loved one** have problems with: (Use **√** to indicate your answer.)	Not at all (**0-1 day**) 0 points	Several Days (**2-6 days**) 1 point	More than half the days (**7-11 days**) 2 points	Almost daily (**12-14 days**) 3 points
Judgment or decision-making				
Repeating the same things over and over such as questions or stories				
Forgetting the correct month or year				
Handling complicated financial affairs such as balancing checkbook, income taxes & paying bills				
Remembering appointments				
Thinking or memory				
Learning how to use a tool, appliance, or gadget				
Planning, preparing, or serving meals				
Taking medications in the right dose at the right time				
Walking or physical ambulation				
Bathing				
Shopping for personal items like groceries				
Housework or household chores				
Leaving her/him alone				
Her/his safety				
Her/his quality of life				
Falling or tripping				
Less interest or pleasure in doing things, hobbies or activities				
Feeling down, depressed, or hopeless				
Being stubborn, agitated, aggressive or resistive to help from others				
Feeling anxious, nervous, tense, fearful or panic				
Believing others are stealing from them or planning to harm them				
Hearing voices, seeing things or talking to people who are not there				
Poor appetite or overeating				
Falling asleep, staying asleep, or sleeping too much				
Acting impulsively, without thinking through the consequences of her/his actions				
Wandering, pacing, or doing things repeatedly				
Over the past **two weeks**, how often did **you** have problems with: (Use √ to indicate your answer.)	Not at all (**0-1 day**) 0 points	Several Days (**2-6 days**) 1 point	More than half the days (**7-11 days**) 2 points	Almost daily (**12-14 days**) 3 points
**Your** quality of life				
**Your** financial future				
**Your** mental health				
**Your** physical health				

Place Sticker Here	**COGNITIVE SUBSCALE**	
	**FUNCTIONAL SUBSCALE**	
	**BEHAVIORAL AND MOOD SUBSCALE**	
	**CAREGIVER STRESS SUBSCALE**	
	**TOTAL SCORE**	

Cut points

Total score: Range: 0 to 81 (total score, sum of 27 items, excludes the 4 QOL items)
- Normal range: ≤14
- Mild symptoms: 15-23
- Moderate symptoms: 24-35
- Severe symptoms: ≥36

Cognitive subscale: Range: 0-18
- Normal range: ≤4
- Mild symptoms: 5-8
- Moderate symptoms: 9-11
- Severe symptoms: ≥12

Functional subscale: Range: 0-33
- Normal range: ≤3
- Mild symptoms: 4-6
- Moderate symptoms: 7-11
- Severe symptoms: ≥12

Behavioral subscale: Range: 0-30
- Normal range: ≤5
- Mild symptoms: 6-7
- Moderate symptoms: 8-11
- Severe symptoms: ≥12

Caregiver stress subscale: Range: 0-12
- Normal range: 0
- Mild symptoms: 1-4
- Moderate symptoms: 5-8
- Severe symptoms: ≥9

A change in the total score of HABC-Monitor of ≥5 indicates a clinically meaningful change.
